# Colorimetric Paper-Based Device for Hazardous Compounds Detection in Air and Water: A Proof of Concept

**DOI:** 10.3390/s20195502

**Published:** 2020-09-25

**Authors:** Valeria De Matteis, Mariafrancesca Cascione, Gabriele Fella, Laura Mazzotta, Rosaria Rinaldi

**Affiliations:** 1 Department of Mathematics and Physics “Ennio De Giorgi”, University of Salento, Via Arnesano, 73100 Lecce, Italy; mariafrancesca.cascione@unisalento.it (M.C.); gabriele93_fella@libero.it (G.F.); ross.rinaldi@unisalento.it (R.R.); 2 Studio Effemme-Chimica Applicata, Via Paolo VI, 73018 Squinzano (LE), Italy; laura.mazzotta@studioeffemme.com

**Keywords:** PAD, environmental monitoring, colorimetric detection, water, atmosphere

## Abstract

In the last decades, the increase in global industrialization and the consequent technological progress have damaged the quality of the environment. As a consequence, the high levels of hazardous compounds such as metals and gases released in the atmosphere and water, have raised several concerns about the health of living organisms. Today, many analytical techniques are available with the aim to detect pollutant chemical species. However, a lot of them are not affordable due to the expensive instrumentations, time-consuming processes and high reagents volumes. Last but not least, their use is exclusive to trained operators. Contrarily, colorimetric sensing devices, including paper-based devices, are easy to use, providing results in a short time, without particular specializations to interpret the results. In addition, the colorimetric response is suitable for fast detection, especially in resource-limited environments or underdeveloped countries. Among different chemical species, transition and heavy metals such as iron Fe(II) and copper Cu(II) as well as volatile compounds, such as ammonia (NH_3_) and acetaldehyde (C_2_H_4_O) are widespread mainly in industrialized geographical areas. In this work, we developed a colorimetric paper-based analytical device (PAD) to detect different contaminants, including Fe^2+^ and Cu^2+^ ions in water, and NH_3_ and C_2_H_4_O in air at low concentrations. This study is a “proof of concept” of a new paper sensor in which the intensity of the colorimetric response is proportional to the concentration of a detected pollutant species. The sensor model could be further implemented in other technologies, such as drones, individual protection devices or wearable apparatus to monitor the exposure to toxic species in both indoor and outdoor environments.

## 1. Introduction

In the last decades, due to the increase of industrialization activities, the release of hazardous materials in the atmosphere, water and soil has raised many concerns about their impact on living organisms [1]. Metals and heavy metals together with gaseous organic compounds are the most widespread toxic elements due to their ability to enter the living organism by different routes, such as inhalation and ingestion [2,3]. Then, they can enter the food chain, integrating into enzymatic processes with the consequence to boost various diseases and inflammation processes onset [4].

Cu(II) and Fe(II) are transition metals having a key role in several physiological pathways, such as fetal growth, brain development, cholesterol metabolism and immune function [5,6,7]. Cu(II) represents one of the main components of the PM 2.5 produced by the road dust emissions, allowing its easy penetration into the organisms’ body [8].

In addition, the ecological risk deriving from Cu(II) exposure is a problem in European saltwater environments [9,10]. Cu(II) can be toxic to aquatic life at concentrations approximately 10 to 50 times higher than the tolerated range [11]. In addition, humans can adsorb a great amount of Cu(II) from drinking water, food, air and supplements, reaching a daily absorption of 1.85 mg [12]. In order to understand the collateral effects of the Cu(II), the US National Toxicology Program (NTP) exposed B6C3F1 mice to the five concentrations of Cu(II) (76, 254, 762, 2543, 7629 mg Cu/L) to [13] for 13 weeks observing the organs weight loss and animals death, at the higher concentrations tested. These results were consistent with another study in which the same toxicity was observed in female and male mice using 762 mg/L of Cu(II). Additionally, Fe(II) triggered adverse effects in vivo by acute toxicity induction [14]. In aquatic environments, Fe(II) boosted the growth default of aquatic organisms at a concentration of 1 mg/L [15]. In addition, in some European countries such as Lithuania, people were exposed to high levels of Fe(II) due to the contamination of groundwater that overcome the permissible limit established by the European Union Directive 98/83/EC, related to the quality of drinking water [16]. Regarding the volatile compounds pollution, NH_3_ is one of the major manufactured industrialized soluble alkaline gases on Earth [17]. NH_3_ originates from both natural and anthropogenic sources, in particular from the agricultural industry, high-density intensive farming practices as well as fertilizer applications [18]. According to the Agency for Toxic Substances and Disease Registry, the concentrations of NH_3_ in the environment are very variable due to its continuous recycling and its internalization in biosphere. Therefore, it is possible to find different natural NH_3_ levels in the soil (1–5 ppm), in air (1–5 ppb) and in water (approximately 6 ppm) [19]. The NH_3_ smell can be identified by humans at concentrations greater than 5 ppm; at 30 ppm and with an exposure time of up to 2 h, human volunteers underwent slight irritation, whereas strong effects were recorded up to 500 ppm [20]. However, NH_3_ lethality requires higher concentrations [21]. In addition to NH_3_**,** also some kinds of carbonyls which constitute the motor vehicle exhaust, such as C_2_H_4_O are toxic air contaminants, particularly dangerous for living organisms [22,23]. Woutersen et al. [24] used Wistar rats to study the toxic effect of C_2_H_4_O administered in air (6 h/day) at three concentrations (750, 1.500, 3.000 ppm) for more than a year. All the concentrations tested induced the increase of nasal tumors incidence with remarkable impact especially at higher concentrations. Other evidences suggested that the C_2_H_4_O administration (1.650–2.500 ppm) for more than two years (7 h/day) induced tracheal, but not nasal, tumors in Syrian golden hamsters [25]. Then, the study of these compounds in polluted areas is a key factor to control the exposure rate.

Today, several analytical techniques are available for the detection of toxic analytes. However, many of them are not affordable due to the expensive instrumentations and high reagent volumes required. On the contrary, point-of-care and easy-to-use analysis provide results in a short time, preventing the production of an elevated amount of waste [26]. In addition, they can be employed in resource-limited environments and developing countries where pollution is uncontrollable and not regulated with specific rules.

In particular, paper is the best choice to develop sustainable devices [27]; it is considered a valid alternative to traditional materials due to its ease of fabrication, satisfactory levels of sensitivity, specificity, low cost, lightweight, versatility, being easily portable and low reagent consumption requiring [28,29]. The paper-based analytical devices (PAD) can work following the principle of color change in the presence of specific target analytes [30]. The sensitivity and specificity of the assay are dependent on an interaction between the target analyte and the surface of the PAD due to the functionalization of cellulose fibers [31]. The paper surface can be functionalized by different molecules, such as chemoresponsive dyes, nanoparticles (NPs) and biomolecules (antibodies, aptamers, nucleic acids) [32,33,34,35]. Xi et al. [36] prepared a paper device based on Pb(II) metal-organic nanotubes characterized by a large {Pb14} metallamacrocycle, to detect H_2_S based on the fluorescence “turn-off” response. However, the fabrication of nanotubes and the general technique required specific scientific competences and elevated costs; moreover the toxicity of nanotubes, is not negligible [37]. Maity et al. [38] used perovskite halide (CH_3_NH_3_PbI_3_) to achieve a thin-film sensor fabricated on a paper by a growth process able to detect NH_3_ gas by a color change from black to yellow. Despite the effectiveness of this device, the H_3_NH_3_PbI_3_ is chemically unstable and toxic for living organisms. [39]. Then, the disposal of the device could present a serious problem. In a recent work [40], a microporous cellulose-based smart xerogel bromocresol purple was used into cross-linked carboxymethyl cellulose to detect NH_3_ by a colorimetric response. The authors performed a freeze-drying process to obtain the xerogel with a low limit of detection.

In these PADs, the colorimetric shift can be evaluated by colorimetric assay, as a result of the interaction with the ligand. In general, the PADs sensing areas are fabricated by the printing method using a wax printer [41]. The results obtained can be directly interpreted by the naked eye together with the spectrophotometer analysis. In the last years, the use of smartphones to detect color change has been developed [42,43,44]. Therefore, its use showed some limitations regarding the low lighting conditions that prevent the smartphone camera exploitation [44].

In this work, we developed an effective PAD suitable to detect different contaminants, namely Fe(II) and Cu(II) cations (Fe^2+^ and Cu^2+^) in water and NH_3_ and C_2_H_4_O vapor in air. The design and fabrication of the sensor did not require specific instrumentations. In particular, for metals detection, only a wax pen able to design the specific areas of chemical interaction was required, without the use of a wax printer. We functionalized the paper (Whatman filter paper) using different analytes capable of reacting with metallic ions and gaseous substances, allowing a specific response; the aim of this process was to develop calibration curves to correlate the obtained color to the concentrations of toxic compounds. The results were easily interpreted using a digital scanner and ImageJ. The tests achieved using intermediate concentrations suggested the sensitivity and reproducibility of the PAD, making it a powerful tool to detect hazardous materials in different mediums without the use of sophisticated technologies.

## 2. Materials and Methods

### 2.1. Ammonia Detection

#### 2.1.1. Reagents

Whatman filter paper n.1 (thickness 180 μm), ammonium hydroxide (NH_4_OH, 28%), hydrochloric acid (HCl), Aniline (C_6_H_5_NH_2_) and ammonium persulfate (NH_4_)_2_S_2_O_8_ were purchased from Merck.

#### 2.1.2. Functionalization of Whatman Paper for Reversible Ammonia Vapor Detection

**Figure 1:**
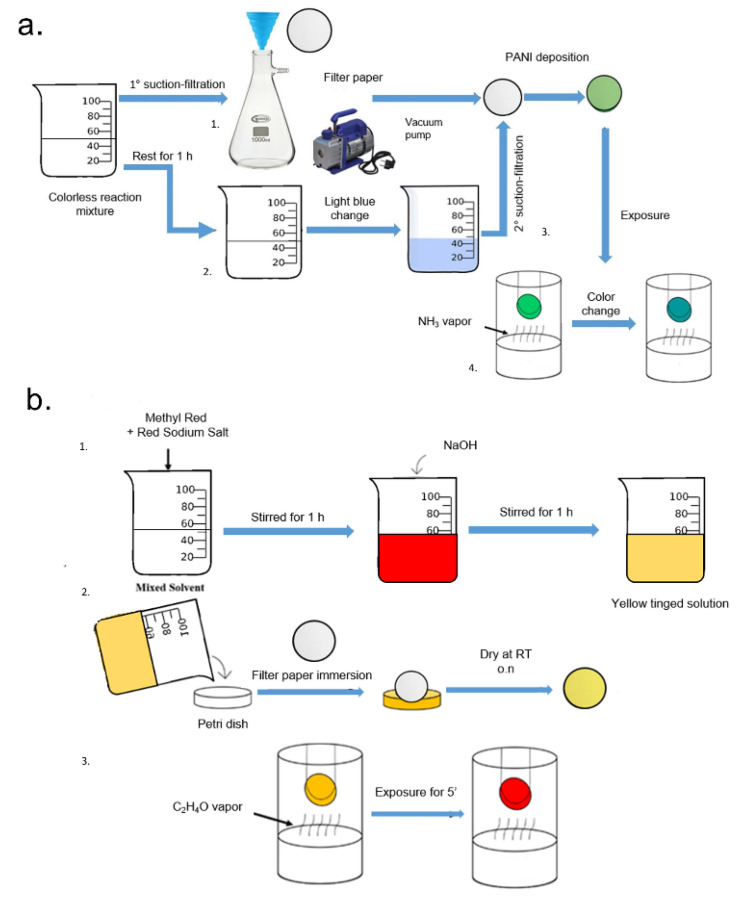
**Schematic NH_3_ (a) and C_2_H_4_O (b) paper sensor fabrication procedure**: (**a**) **1**. Half of the colorless reaction mixture was immediately suction-filtered into a silicon funnel where a filter paper (*white circle*) was placed. **2.** The remaining part of the solution was left to stand for 1 h. During this time, the solution turned light blue. **3.** The solution was filtered (II suction-filtration) through the filter paper, in order to induce the polyaniline (PANI) deposition. After several washes and air flow drying, the formation of emerald green filter paper (*green circle*) was completed. **4**. The emeraldine green filter paper turned into a blue emeraldine base (*blue circle*) as a result of NH_3_ vapor exposure. (**b**) **1.** The methyl red and methyl red sodium Salt were added to the mixture. The color solution turned into red-orange and was stirred for 1 h. After this time, NaOH was added, resulting in a color change to yellow. The solution was left to stand for 1 h. **2**. The solution was transferred in a petri dish and the filter paper was immersed in it for 1 h. The filter paper was dried overnight in the dark at room temperature. The formation of methyl red filter paper (*yellow circle*) was completed. **3**. The methyl red filter paper turned into red (*red circle*) as a result of C_2_H_4_O vapor exposure.

The reversible colorimetric detection of gaseous NH_3_ was realized by coating Whatman filter paper with polyaniline (PANI) film, achieved by C_6_H_5_NH_2_ polymerization (2.5 g/L) in the presence of HCl (1 M) and (NH_4_)_2_S_2_O_8_ (0.125 g/L) at room temperature [45]. Briefly, (NH_4_)_2_S_2_O_8_ solution was added dropwise into the C_6_H_5_NH_2_ solution under stirring (1000 rpm). The two compounds were in a volume ratio of 1:1. After 3 min, half of the colorless reaction mixture was immediately added into a silicon funnel, where a piece of round filter paper (c.a 2 cm) was placed and fixed. Then, the remaining solution was slowly suction-filtered through the filter paper, and the unused volume was left in the dark for approximately 1 h. During this time, the solution slowly turned light blue. After this step, the solution was again filtered and then, the paper was carefully washed with Milli-Q water. Finally, it was left to air dry until the emerald green filter paper was achieved. The functionalized paper was exposed to different concentrations of NH_3_. The schematic representation of this procedure is represented in Figure 1a.

#### 2.1.3. Construction of Calibration Curve by Colorimetric Response to Ammonia Vapor

Glass vials were used to detect NH_3_ vapor exposure. In each vial, 10 mL of NH_3_ solution was added at different concentrations (100, 300 500 and 1000 ppm) to achieve a standard curve. Small PANI-deposited filter paper pieces were fixed on the necks of the vials in order to expose them to the vapor generated from the corresponding NH_3_ aqueous solution for a few seconds. The control was represented by pure NH_3_. After this time, the paper was immediately removed and analyzed by a scanner (Samsung SCX-3400 series (USB002)) acquiring the color change after NH_3_ vapor interaction.

### 2.2. Acetaldehyde Detection

#### 2.2.1. Reagents

Whatman Filter paper n.1 (thickness 180 μm), methyl red (C_15_H_15_N_3_O_2_), methyl red sodium salt (C_15_H_14_N_3_NaO_2_), methanol (CH_3_OH), Glycerol (C_3_H_8_O_3_) and sodium hydroxide (NaOH) were purchased from Merck.

#### 2.2.2. Functionalization of Whatman Paper for Acetaldehyde Vapor Detection

The colorimetric detection of gaseous C_2_H_4_O was obtained by coating a Whatman filter paper with thin methyl red film. methyl red and methyl red sodium salt was dissolved in a solvent constituted by CH_3_OH, water and C_3_H_8_O_3_ (1 mM). The red-orange solution was stirred for approximately 1 h. NaOH (8 mM) was added to the solution and stirred at room temperature for 1 h. The yellow-colored solution obtained was translocated in a petri dish where a piece of Whatman filter paper was immersed for 1 h. After this time, the paper was dried at room temperature overnight. The filter paper sheet was then cut into small round disks (diameter of approximately 2 cm) and successively exposed to different concentrations of C_2_H_4_O. After 5 min, the color appeared on the paper. The schematic representation of this procedure is represented in Figure 1b.

#### 2.2.3. Construction of Calibration Curve by Colorimetric Response to Acetaldehyde Vapor

The C_2_H_4_O vapor detection was performed using different glass vials in which 10 mL of C_2_H_4_O was added at different concentrations in CH_3_OH solvent: 100, 300, 500 and 1000 ppm, respectively, on the vial’s neck. The deposited filter paper pieces were fixed in order to expose them to the vapor evaporated from each C_2_H_4_O/CH_3_OH solution for 5 min. After this time, the paper was immediately removed and analyzed by a scanner (Samsung SCX-3400 series (USB002)) in order to acquire the color changes after C_2_H_4_O interaction.

### 2.3. Fabrication of Paper-Based Colorimetric Device for Fe^2+^ and Cu^2+^

#### 2.3.1. Reagents

Iron chloride tetrahydrate (FeCl_2_·4H_2_O), HCl, copper sulfate pentahydrate (CuSO_4_·5H_2_O), potassium ferricyanide (K_3_[Fe(CN)_6_]), and potassium iodide (KI) were purchased from Merck.

#### 2.3.2. Iron and Copper Calibration Curve Standard Solutions Preparation

FeCl_2_·4H_2_O was dissolved in HCl (0.5 M) in order to achieve 1000 μg/mL of Fe^2+^ standard stock solution whereas CuSO_4_·5H_2_O was used to prepare 1000 μg/mL Cu^2+^ standard stock solution in Milli-Q water. The series of four standard solutions (25, 50, 100 and 200 μg/mL) of Fe^2+^ and Cu^2+^ were prepared by diluting the standard stock solutions with different volumes of Milli-Q water. After these steps, K_3_[Fe(CN)_6_] (5 mM) and KI (0.4 M) solutions were prepared for Fe^2+^ and Cu^2+^ detection, respectively.

#### 2.3.3. Fabrication of the Paper Analytical Device (PAD)

The fabrication of PAD was developed as follows:

1. The waxy channels on a piece of Whatman filter paper were obtained by using a wax pen. The shape of each channel was circular with a diameter of approximately 0.5 cm. Four spots were drawn on the filter paper, one for each standard.
2. The PAD was heated on a hot plate at ~60 °C for 1 h to melt the wax. The liquid wax penetrated into the cellulose pores to achieve hydrophobic barriers.
3. The PAD was dried at room temperature for approximately 30 min.

#### 2.3.4. Assay Procedure

**Figure 2:**
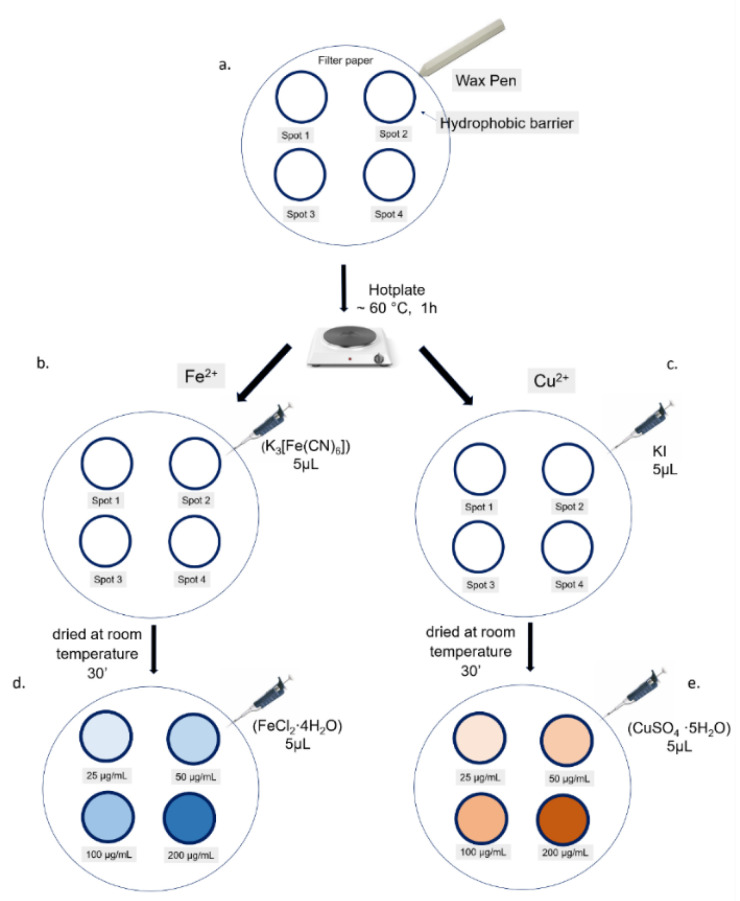
**Schematic Fe^2+^ and Cu^2+^ colorimetric assay procedure**: (**a**) The four spots were achieved by wax pen in order to create hydrophobic barriers after heating using a hot plate. (**b**,**c**) Five microliters of each standard solution were added by drop-casting to the corresponding labeled spot. (**d**) On the Fe(II) paper-based analytical device (PAD), a blue complex was formed after the reaction between the Fe^2+^ and ((K_3_[Fe(CN)_6_]); the blue color intensity directly correlated with the Fe^2+^ concentration (**e**) On the Cu(II) PAD a red-brown compound was developed, generating by Cu^2+^ and KI reaction, whose color intensity was dependent on Cu^2+^ concentration.

A small volume (5 μL) of Fe^2+^ and Cu^2+^ assay reagents ((K_3_[Fe(CN)_6_]) and KI) was spotted by drop-casting on paper circular dots using a micropipette and allowed to dry at the room temperature for 3 h. Five microliters of each standard solution was added to the corresponding labeled spots of the PAD. The Fe^2+^ of the standard solutions reacted with the K_3_[Fe(CN)_6_] generating blue colored complex in the detection zones. Instead, the Cu^2+^, reacting with KI, produced a red-brown compound. The intensity of the color was proportional to the standard solution concentration. A schematic representation of the described process was represented in Figure 2.

### 2.4. Quantitative Image Processing by ImageJ 1.47 Software

Once the color changes were achieved due to the chemical interaction with the different hazardous compounds, the corresponding PADs images were captured using scanner Samsung SCX-3400 with a resolution of 300 dpi. Then, the images were stored in JPEG format and analyzed in RGB format with the open-source software, ImageJ [46]. An adjustment of the color threshold was applied to each image to filter out all colors that were not correlated to the colored complex to be detected during the analysis. For instance, the Fe^2+^ color adjustment was applied to delete all colors which was not in the blue range from the analysis spectrum. The color adjustment was set as follows:

1. The “Color Threshold” window was accessed through the ImageJ menu by selecting “Image”→ “Adjust”→“Color Threshold.”
2. At the bottom of this window HSB was selected, which allowed the adjustment of hue, saturation, and brightness.
3. The hue was adjusted by moving the sliders directly below the “Hue” spectrum until only the color of interest was visible. The hue threshold ranges set for each metal were fixed as follows: NH_3_ (244–255), C_2_H_4_O (38–240), Fe^2+^ (171–197), Cu^2+^ (37–255).

The images were then converted to an 8-bit grayscale (“Image” → “Type” → “8-bit”) and inverted (“Edit” → “Invert”). The intensity measurements yielded a positive slope when plotted versus metal amounts. Mean Gray Value (MGV) was measured for each RGB channel (red, blue and green, “Image” → “Color” → “Merge Channel”) by first selecting “mean gray value” and “limit to threshold” in the “Set measurements window,” found from the ImageJ menu by selecting “Analyze” → “Set measurements”. Each area was selected using the wand tool, which automatically found the edge of an object and traced its shape. The gray intensity of the outlined area was measured by selecting “Analyze” → “Measure.” Then, the RGB channel was selected with the highest sensitivity for the metal detection according to Yu et al. [47]. The blue channel was selected for both metal cations¸ the red channel for NH_3_ and green channel for C_2_H_4_O were selected. Data were then imported into Microsoft Excel 2019 in order to obtain the different calibration curves for the NH_3_, C_2_H_4_O, Fe^2+^, Cu^2+^ concentrations.

The colorimetric detection limits of NH_3_, C_2_H_4_O, Fe^2+^ and Cu^2+^ were estimated based on 3SB/S according to IUPAC rules, where SB and S are standard deviation and slope, respectively [48,49].

### 2.5. Interference Studies

The selectivity of PAD to Cu^2+^ and Fe^2+^ was evaluated by interferences assessment exposing the functionalized PAD to several metal ions solutions containing Na^+^, K^+^, Mg^2+^, Ca^2+^, Al^3+^, Mn^2+^, Fe^3+^, Co^2+^, Ni^2+^, Zn^2+^, Cd^2+^ and Pb^2+^ at a concentration of 100 μg/mL. The same procedure was used to assess the specificity of PAD to NH_3_ and C_2_H_4_O using methylamine, ethylamine, triethylamine, benzene, toluene, ethyl benzene, formaldehyde and ethanol at a concentration of 500 ppm.

## 3. Results and Discussion

**Figure 3:**
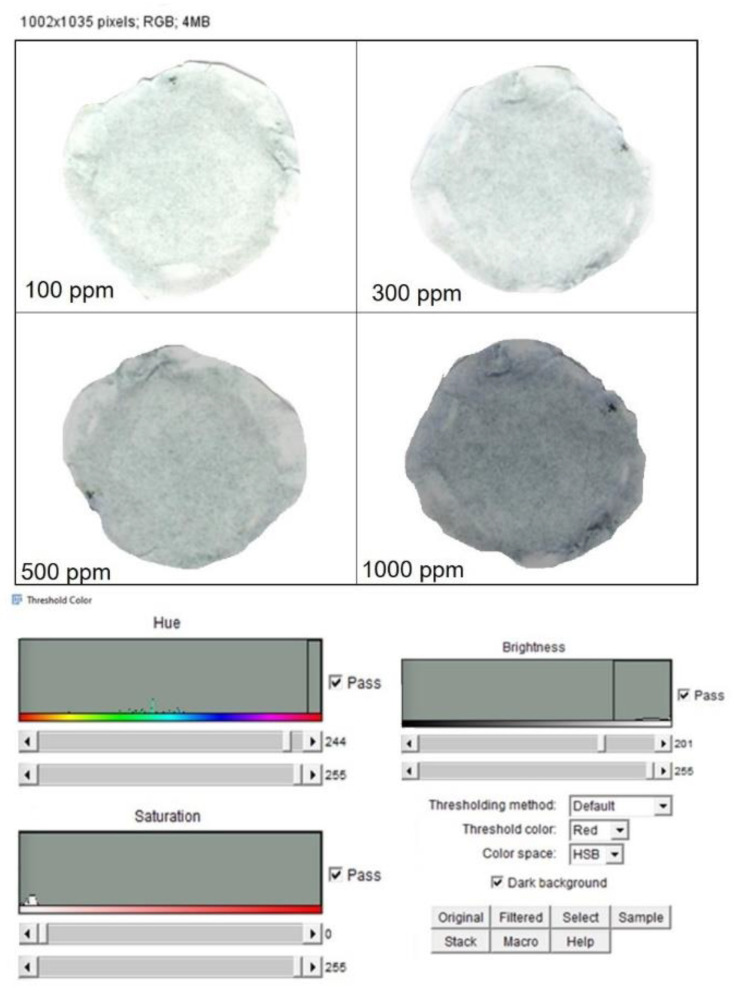
Hue adjustment section of the Threshold Color window in ImageJ analysis software of the NH_3_ PAD.

In recent years, the environmental pollution has been at the center of many debates, due to the progressive and intense industrialization; the scientific community has thus focused its attention on the potentially toxic effects of certain substances on the living organisms [50]. Several people are exposed to different kinds of substances owing to the contamination of several environments in particular water, atmosphere and soil [51]. Among these, the most widespread are certainly the transition metals, heavy metals and gaseous substances, that are produced by intense processing activities especially in the agrifood sector [52]. These chemicals are generally released into the atmosphere and they can reach the groundwater as well as lakes and sea reaching living organisms with subsequent collateral effects [3,53]. In this scenario, environmental monitoring is a fundamental objective to prevent and to know at what doses an organism was exposed. The conventional analytical techniques (gas chromatography–mass spectrometry, high-performance liquid chromatography–mass spectrometry, atomic absorption spectroscopy) are sophisticated systems that require high energy consumption and expensive laboratory systems. Paradoxically, in fact, the environment analysis by the use of these instruments induces in turn pollution (energy, consumables, toxic reagents). Starting from these assumptions, we have developed a PAD that can be used without the need for trained operators to monitor some hazardous materials such as NH_3_, C_2_H_4_O, Fe^2+^ and Cu^2+^. For gaseous substances, namely NH_3_ and C_2_H_4_O, we performed two different techniques to functionalize the filter paper. In particular, for NH_3_ detection, we used a PANI film functionalization following the polymerization of aniline directly on paper substrate. The PANI film obtained was in the form of green emeraldine salt due to the protonation of the backbone induced by HCl. We selected four doses of NH_3_ on the basis of toxicological results obtained in literature, as reported in the Introduction section (Section 1). When NH_3_ molecules reached the functionalized paper, the deprotonation of PANI chains and, consequently, the transformation of them into a blue emeraldine base occurred. In addition, this dye shows peculiar chemical properties consisting of the reversible doping/dedoping nature. The dye reacted with the NH_3_ determining the color change; when the analyte was removed, it can be reverted to its initial chemical state. Due to the reversible nature of the process, the functionalized PAD can be reused many times (ca. 30 times) before its discard. After the exposure to different concentrations of NH_3_ vapor (100, 300, 500, 1000 ppm) the color appeared in a few minutes. Immediately, a digital scanner was used to freeze the specific color. The scanner acquired the image in JPEG format, allowing the next analysis by ImageJ software. As shown in Figure 3, the paper assumed a specific coloration that can be visualized with the naked eye. The color switch from light green to blue at the higher concentration tested. By The JPEG images were analyzed after setting the specific parameters (Hue adjustment section of the Threshold Color window) described in detail in the Materials and Methods section (Section 2). The assay reproducibility was evaluated for three identical test zones.

**Figure 4:**
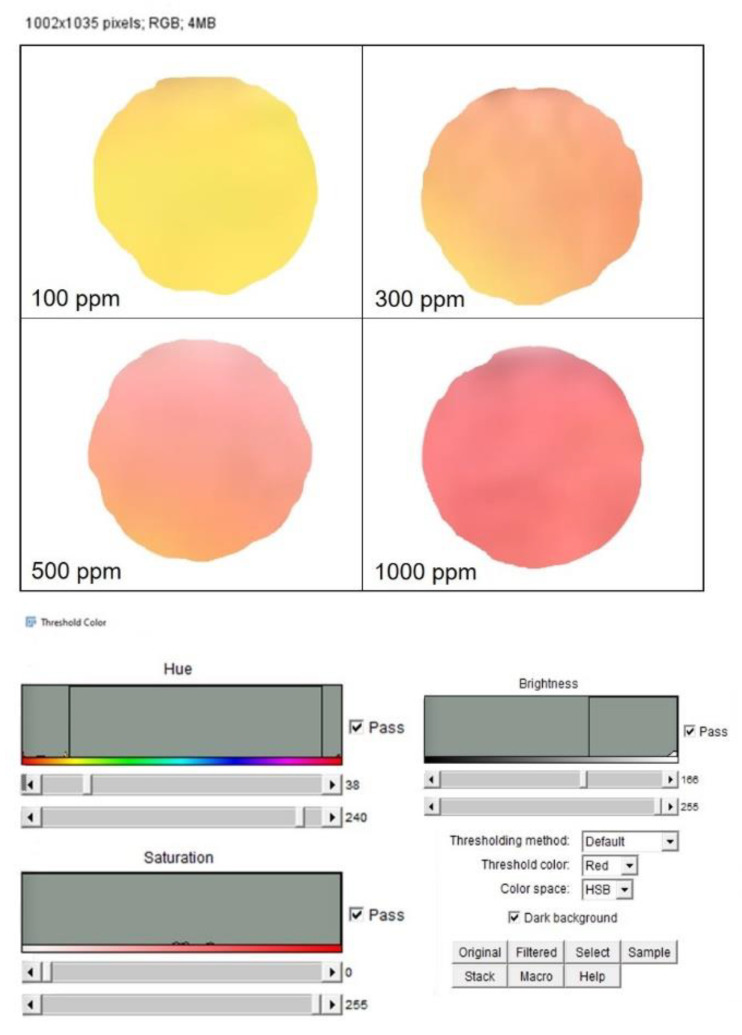
Hue adjustment section of the Threshold Color window in ImageJ of C_2_H_4_O PAD.

The functionalization of PAD for the detection of C_2_H_4_O was achieved by the use of methyl red. The latter is determined by the concentration of acidic (red) and basic (yellow) forms. The colorimetric sensor was designed to show a selective response based on a chemical reaction, such as the nucleophile addition. Using an excess of hydroxide ions, the C_2_H_4_O underwent the nucleophile addition reaction, resulting in the sensor alkalinity changes and consequently in a color change, from yellow to red. The color change was almost instantaneous and it was stable for several days after drying. After the color response, the scanner was used to acquire the image and color intensity. The latter was analyzed for the second step of the experimental session using ImageJ analysis by the Hue adjustment section of the Threshold Color (Figure 4). The reproducibility was evaluated for three identical test zones.

**Figure 5:**
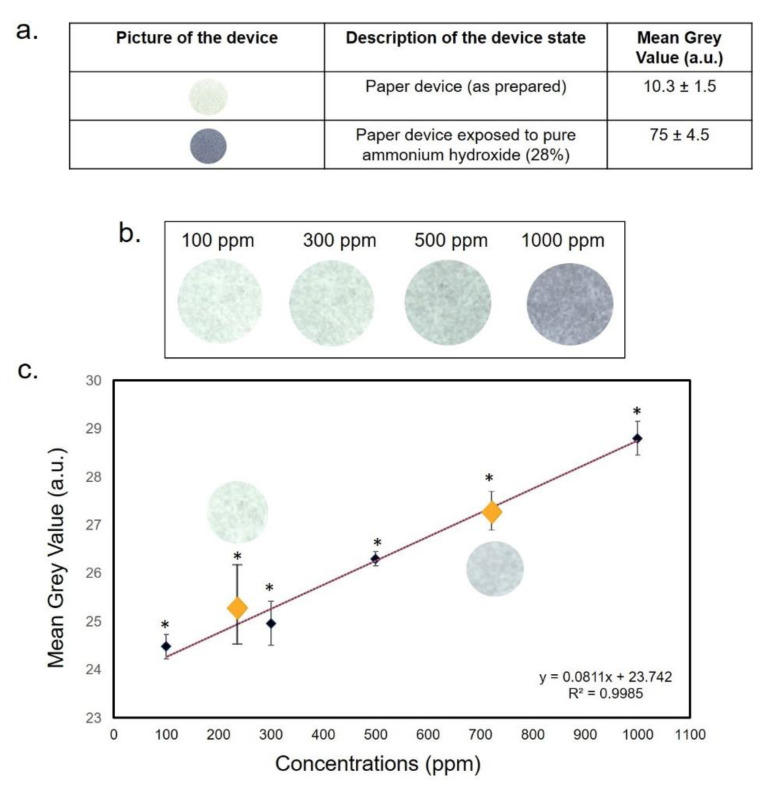
(**a**) Mean Gray Value (MGV) values of PAD as prepared and after the exposure to pure NH_3_ (28%). (**b**) color change after NH_3_ exposure. (**c**) Interpolation of NH_3_ intermediate values (200 and 750 ppm). Data reported were the average of three independent experiments ± SD. The difference between the as-prepared paper and colored papers was considered statistically significant performing a Student’s *t*-test with *p* < 0.05 (<0.05 *).

The test zones were used to create the calibration curve. Figure 5 shows the calibration curve for NH_3_ detection using the color change after the exposure to the four concentrations. In detail, in Figure 5a we reported the pieces of devices related to the functionalized and unexposed PAD (top circle) and the PAD exposed to pure NH_3_ (28%, bottom circle) with the relative MGV values extracted from the ImageJ analysis that were 10.3 ± 1.5 and 75 ± 4.5, respectively. In Figure 5b, the pieces of PAD after exposure to 100, 300, 500 and 1000 ppm of NH_3_ were represented. Observing the pictures, it was possible to visualize a color trend with the naked eye, from the lightest to the darkest as the concentration increased. The successive ImageJ analysis performed on the scanner acquisitions correlated with the concentration with a specific MGV obtaining a calibration curve with R^2^ = 0.99. The limit of detection (LOD) value was 7.64 ppm. The values were obtained by repeating the experiment three times. In order to understand if the device actually worked even with intermediate concentrations, we exposed the PAD to average concentrations calculated between the first and second (200 ppm) and third and fourth doses (750 ppm). Additionally, in this case, PANI film was able to efficiently induce the color response; it was possible to find the concentration simply by interpolating the MGV data on the straight line as shown in Figure 5c.

**Figure 6:**
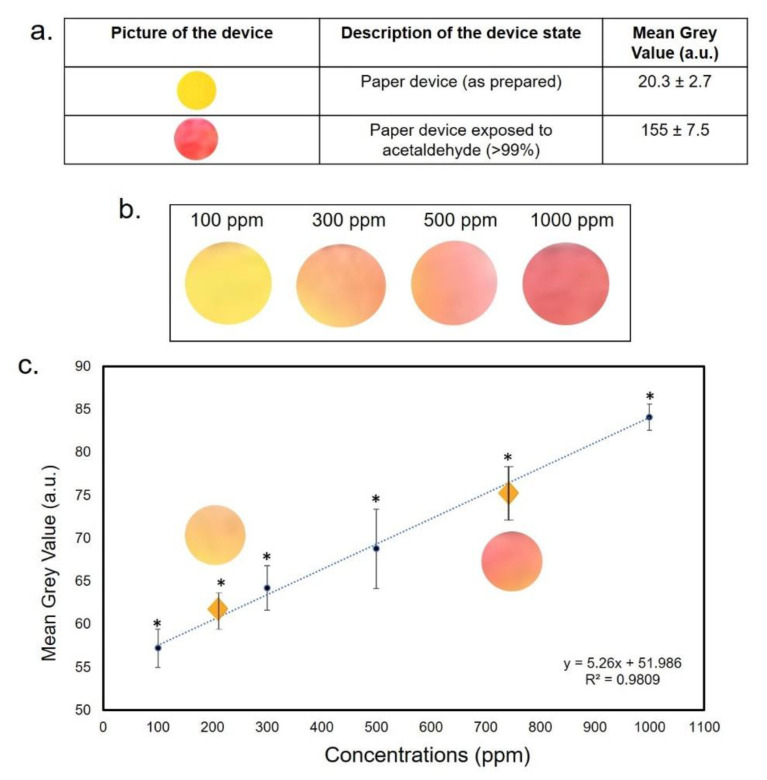
(**a**) MGV values of PAD as-prepared and after the exposure to pure C_2_H_4_O (>99%). (**b**) color change after C_2_H_4_O exposure. (**c**) Interpolation of C_2_H_4_O intermediate values (200 and 750 ppm). Data reported were the average of three independent experiments ± SD. The difference between the as-prepared paper and colored papers was considered statistically significant performing a Student’s *t*-test with *p* < 0.05 (<0.05 *).

The same procedure was applied to the paper functionalized with methyl red, capable of detecting the C_2_H_4_O vapor. (Figure 6). Figure 6a shows the as-prepared paper device (yellow) and after exposure to pure C_2_H_4_O (≥ 99.5%, dark red) with the corresponding MGV values that were 20.3 ± 2.7 and 155 ± 7.5, respectively. In Figure 6b, the progression from yellow to red was observed when the tested concentrations increased. We used 100, 300, 500 and 1000 ppm concentrations and we built the calibration curve obtaining an R^2^ value of 0.98 (Figure 6c). The LOD value was 11.09 ppm. The effectiveness of this colorimetric response was verified using two average concentrations: 200 and 750 ppm. Additionally, in this case, the MGV values were interpolated with the curve that exactly corresponded to the tested concentrations.

After the analysis of gaseous molecules, we used the PAD to detect Cu^2+^ and Fe^2+^, which are the most common metals released in the environment [1]. Then, we moved to the detection of these metals in water at low concentrations. Firstly, we designed four circle spots using a wax pen in order to achieve hydrophobic barriers without the use of wax printing, inkjet printing and screen-printing technologies. Once the heat produced by the hot plate allowed the penetration of the wax into the cellulose porous, the specific chemical analytes, K_3_[Fe(CN)_6_] for Fe^2+^ and KI for Cu^2+^, were deposited in the spot’s center by drop-casting. The wax channels prevented the typical diffusion phenomenon of the liquid substances deposited on the paper. Five microliters of FeCl_2_.4H_2_O and CuSO_4_.5H_2_O (25, 50, 100, 200 µg/mL) were used. Chelation (1) and redox (2) chemical reactions produced a blue and brown color, respectively. The two reactions were the following:

(1) Fe^2+^ + Fe(CN)^3−^_6_ → Fe_3_[Fe(CN)_6_]^2^;

(2) Cu^2+^ + 2I^−^ →CuI_2_ → 2CuI_2_ = 2CuI + I_2_.

**Figure 7:**
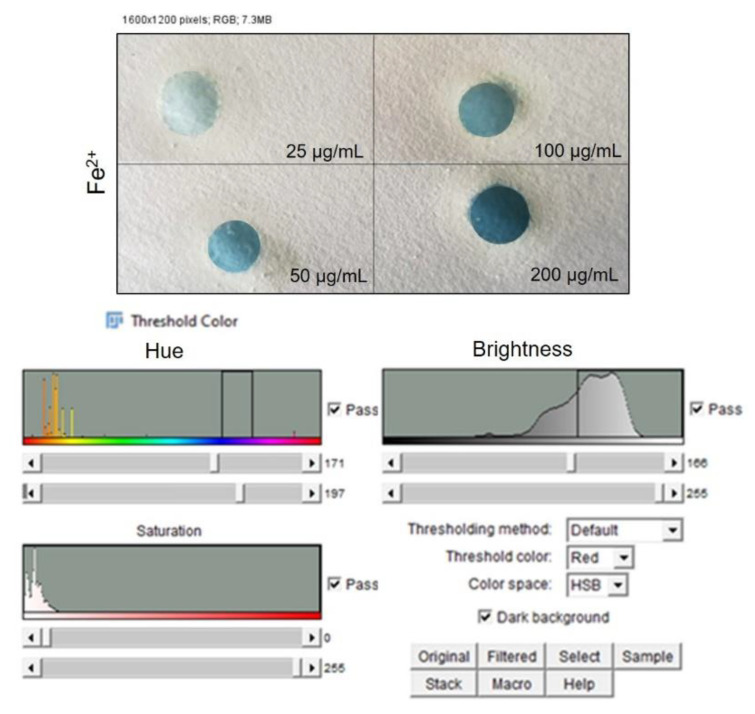
Top: Image acquisition of PAD after FeCl_2_.4H_2_O deposition at different concentrations. Down: threshold analysis, saturation and brightness adjustment by ImageJ software.

**Figure 8:**
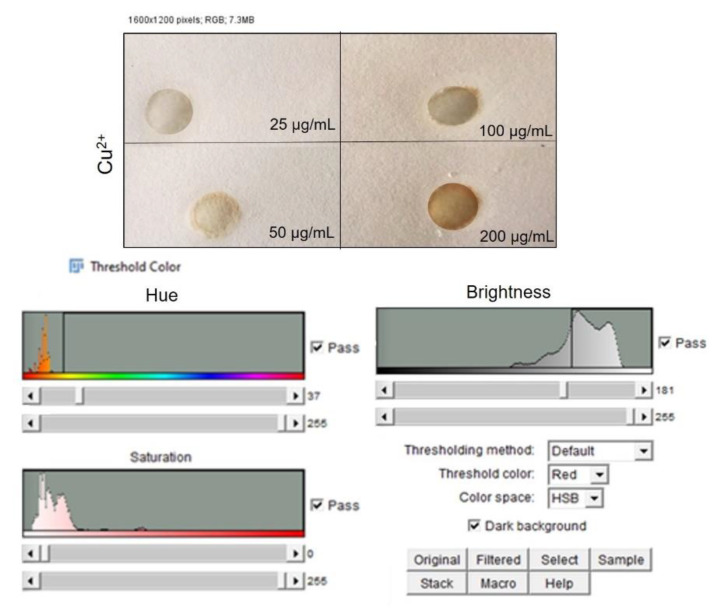
Top: Image acquisition of PAD after CuSO_4_.5H_2_O deposition at different concentrations. Down: threshold analysis, saturation and brightness adjustment by ImageJ software.

After color formation, we acquired the images by a digital scanner to perform ImageJ analysis using the adjustment of Threshold Color both for Fe^2+^ (Figure 7) and Cu^2+^ (Figure 8). The analysis was repeated in three identical test zones.

**Figure 9:**
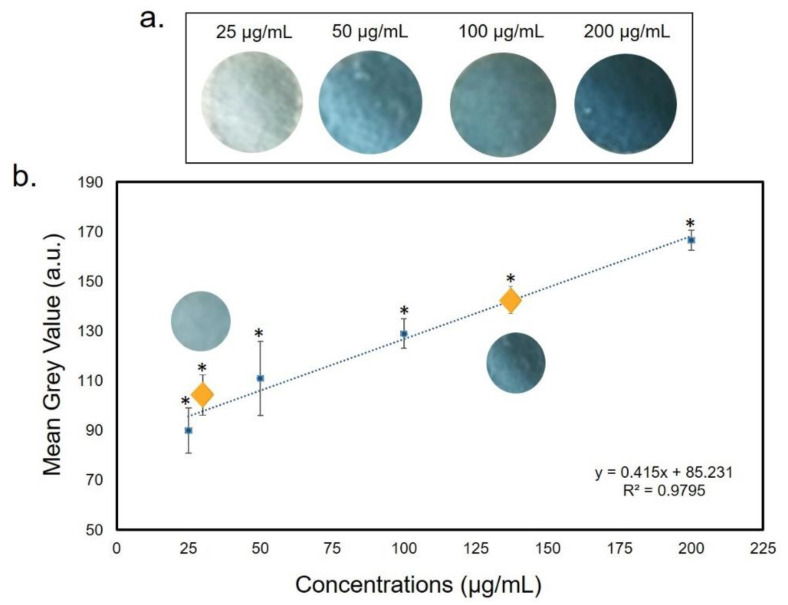
(**a**) Color change of filter paper after exposure to Fe(II) at different concentrations. (**b**) The yellow rhombuses represented the interpolation of Fe^2+^ intermediate concentrations (37 and 150 µg/mL). Data reported were the average of three independent experiments ± SD. The difference between as-prepared paper and colored papers was considered statistically significant performing a Student’s *t*-test with *p* < 0.05 (<0.05 *).

As shown in Figure 9a, the color changed from light blue to dark blue, proportionally to the Fe^2+^ concentration increase. The corresponding calibration curve was obtained plotting the MGV values analyzed by ImageJ analysis after standards solution deposition, showing an R^2^ value of 0.98 (Figure 9b).

**Figure 10:**
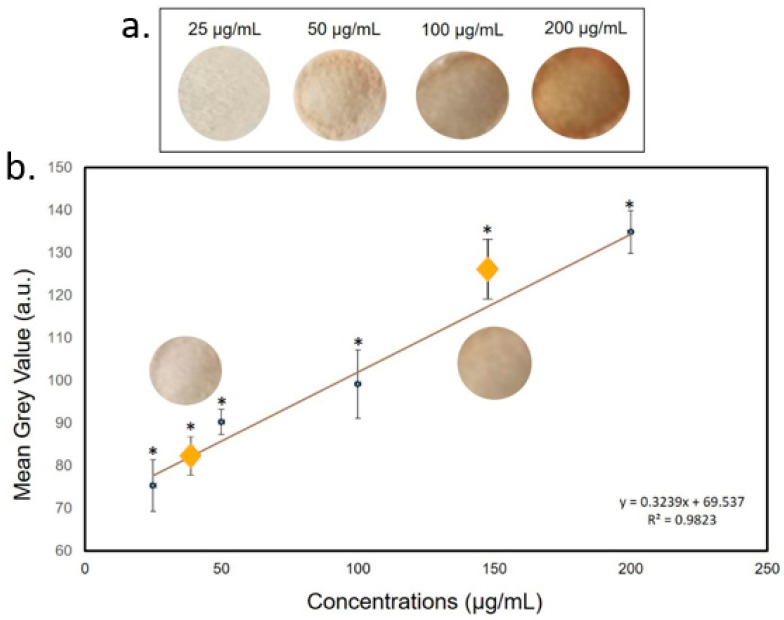
(**a**) Color change of filter paper after exposure to Cu(II) at different concentrations. (**b**)The yellow rhombuses represented the interpolation of Cu^2+^ intermediate concentrations (37 and 150 µg/mL). Data reported were the average of three independent experiments ± SD. The difference between as-prepared paper and colored papers was considered statistically significant performing a Student’s *t*-test with *p* < 0.05 (<0.05 *).

A similar R^2^ value was reported for the Cu^2+^ calibration curve; in the latter case, the color changed from light brown to dark brown (Figure 10a). As demonstrated for NH_3_ and C_2_H_4_O we used two average concentrations between 25 and 50 µg/mL and between 100 and 200 µg/mL (37 and 150 µg/mL) to test the device reliability. The MGV values acquisitions revealed that the corresponding concentrations were on the calibration curve thus confirming the effectiveness and stability of the PAD (Figure 10b). The LOD for Fe^2+^ was 3.8 µg/mL and 3.2 µg/mL for Cu^2+^. For both metals, the values were greatly below the maximum acceptable concentrations in drinking water stipulated by the World Health Organization (WHO) [54].

The LOD values of each device are summarized in Table 1.

**Figure 11:**
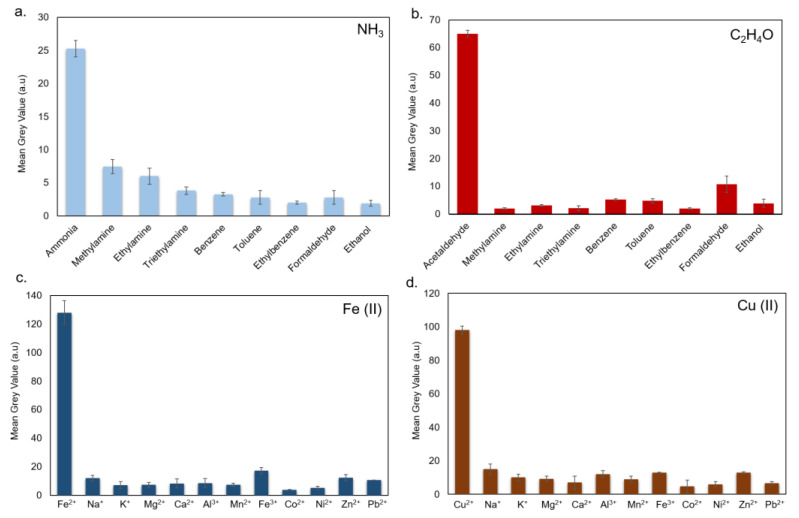
Interferences assay for NH_3_ and C_2_H_4_O (**a**,**b**) and Fe(II) and Cu (II) (**c**,**d**). The values were expressed as MGV. Data reported were the average of three independent experiments ± SD.

In order to test the selectivity of the different PADs used in this study, several metal and gaseous solutions at 100 µg/mL and 100 ppm, respectively, were used. No significant visual color change had been observed in all the tested cases. For gaseous molecules, the PAD was exposed to methylamine, ethylamine, triethylamine, benzene, toluene, ethyl benzene, formaldehyde and ethanol at 100 ppm for ca. 15 min. Any noticeable effects on filter paper were recorded. This suggested the high selectivity of PAD to the NH_3_ and C_2_H_4_O only (Figure 11a,b). Similar results were obtained analyzing the interferences of different metal ions after PAD exposure for 15 min. It was observed that 100 μg/mL of Na^+^, K^+^, Mg^2+^, Ca^2+^, Al^3+^, Mn^2+^, Fe^3+^, Co^2+^, Ni^2+^, Zn^2+^, Cd^2+^, and Pb^2+^ highlighted negligible colorimetric effects on the PAD due to the small affinity with the analytes deposited on filter paper (Figure 11c,d).

## 4. Conclusions

The use of paper as a platform for sensing devices offers considerable advantages in terms of affordability and availability of functionalization processes; in fact, the hydrophilic nature of paper makes it a suitable tool due to the fast adsorption of different chemical solutions through its porous structure. Since only small volumes of reagents are needed to functionalize the paper device, it is very inexpensive. In addition, this technology does not require qualified personnel to collect and analyze the data. We developed an easy and versatile PAD that is able to measure different pollutant agents, namely NH_3_, C_2_H_4_O, Fe^2+^ and Cu^2+^, in two different mediums, air and water. The device architecture is a “proof of concept” of a new class of colorimetric sensors. In fact, it could be implemented in several environmental detection technologies, such as drones or aquatic sensors as well as individual protection devices or wearable technologies, by an electronic integration. In addition, the PAD can be used by different citizens of particular geographic areas to map the possible contaminations, with the aim to collect the global data and to build a database to monitor the pollution.

## Figures and Tables

**Table 1:** LOD values of NH_3_, C_2_H_4_O, Fe^2+^ and Cu^2+^ PADs.

PADs	Concentrations Range	Limit of Detection (LOD)
NH_3_	100–1000 ppm	7.64 ppm
C_2_H_4_O	100–1000 ppm	11.08 ppm
Fe^2+^	25–200 µg/mL	3.8 µg/mL
Cu^2+^	25–200 µg/mL	3.2 µg/mL
